# Quantifying App Store Dynamics: Longitudinal Tracking of Mental Health Apps

**DOI:** 10.2196/mhealth.6020

**Published:** 2016-08-09

**Authors:** Mark Erik Larsen, Jennifer Nicholas, Helen Christensen

**Affiliations:** ^1^ Black Dog Institute University of New South Wales Sydney Australia; ^2^ School of Psychiatry University of New South Wales Sydney Australia

**Keywords:** mobile applications, mobile apps, mental health, telemedicine, depression, bipolar disorder, suicide

## Abstract

**Background:**

For many mental health conditions, mobile health apps offer the ability to deliver information, support, and intervention outside the clinical setting. However, there are difficulties with the use of a commercial app store to distribute health care resources, including turnover of apps, irrelevance of apps, and discordance with evidence-based practice.

**Objective:**

The primary aim of this study was to quantify the longevity and rate of turnover of mental health apps within the official Android and iOS app stores. The secondary aim was to quantify the proportion of apps that were clinically relevant and assess whether the longevity of these apps differed from clinically nonrelevant apps. The tertiary aim was to establish the proportion of clinically relevant apps that included claims of clinical effectiveness. We performed additional subgroup analyses using additional data from the app stores, including search result ranking, user ratings, and number of downloads.

**Methods:**

We searched iTunes (iOS) and the Google Play (Android) app stores each day over a 9-month period for apps related to depression, bipolar disorder, and suicide. We performed additional app-specific searches if an app no longer appeared within the main search

**Results:**

On the Android platform, 50% of the search results changed after 130 days (depression), 195 days (bipolar disorder), and 115 days (suicide). Search results were more stable on the iOS platform, with 50% of the search results remaining at the end of the study period. Approximately 75% of Android and 90% of iOS apps were still available to download at the end of the study. We identified only 35.3% (347/982) of apps as being clinically relevant for depression, of which 9 (2.6%) claimed clinical effectiveness. Only 3 included a full citation to a published study.

**Conclusions:**

The mental health app environment is volatile, with a clinically relevant app for depression becoming unavailable to download every 2.9 days. This poses challenges for consumers and clinicians seeking relevant and long-term apps, as well as for researchers seeking to evaluate the evidence base for publicly available apps.

## Introduction

For many mental health conditions, the ability to deliver information, support, and intervention outside the clinical setting is a major advantage. The growing ubiquity of smartphones is increasingly making this possible, as a recent report indicated that 79% of smartphone users are with their phones for all bar 2 of their waking hours [1]. Recent surveys suggest that 25% of adults use mobile apps for health care [2], and 71% of patients in an outpatient psychiatric setting indicated a desire to use an app to supplement their clinical care [3]. Moreover, in the United States, a third of clinicians reported having recommended a health care app within the last year [4].

This use of mobile apps for health care is largely consumer led and commercially driven. App developers rate app stores as the preferred distribution channel of health apps, rather than through physicians or hospitals, and this distribution is likely to continue until at least 2020 [5]. However, there are difficulties with the use of a commercial marketplace to distribute health care resources.

App store descriptions offer little information about app content quality and rarely cite the source of their content or substantiate claims of effectiveness. A review of the former UK National Health Service (NHS) Health Apps Library found that, of the mental health apps accredited by the NHS, only 15% provided evidence of effectiveness [6]. Previous studies have also identified a lack of research-based evidence associated with mental health apps generally [7], and mood disorders specifically [8]. Furthermore, a growing number of studies have identified a disparity between app content and evidence-based practice [9-11]. This highlights the challenge for consumers and clinicians in selecting mobile health apps from the app stores.

A recurring limitation of these evidence reviews is that they only constitute a snapshot of a highly dynamic marketplace. The systematic review methodologies used ensure rigor; however, compared with publications in the academic literature, additions to the app stores are frequent, and removal of apps is common. There are several possible reasons for apps to be removed, including decisions made by the developer, nonrenewal of a developer account, and withdrawal by the app store operator. If apps are to be used by consumers and clinicians, app longevity is an important consideration. To date, to our knowledge, there has been no systematic investigation of the dynamics of the app stores, in particular app turnover.

Therefore, the overall purpose of this study was to provide a better understanding of the app store environment, with a specific focus on apps for mental health. To achieve this, the primary aim was to establish two metrics for the official Android and iOS app stores: the period of time after which 50% of apps identified by a specific keyword search changed and no longer appeared in the search results (the *search result half-life*); and the period of time after which 50% of apps identified in an initial search were no longer available to download (the *app half-life*). The turnover represented by the search result half-life is relevant to users broadly searching for apps using a keyword search and represents the rate of change of these search results. The app half-life metric is relevant for users searching for a specific app, for example, by following a direct link to the app’s page on the app store, and provides an indication of the longevity of an app being available to download, irrespective of its inclusion in the search results (eg, due to decreasing popularity). The secondary aim of this study was to identify the proportion of clinically relevant apps identified in the search, and to compare the longevity of these apps with that of the clinically nonrelevant apps. The tertiary aim was to identify the proportion of clinically relevant apps that substantiated claims of clinical effectiveness.

Finally, we preformed supplementary analyses to explore the factors that are publicly visible on the app stores, which may affect the search result half-life and the app half-life. These factors include app search result ranking, star rating, and number of downloads. A better understanding of this largely unregulated space will illuminate some of the challenges of mobile health (mHealth) providing valuable health care resources in an uncertain environment.

## Methods

### Data Collection

We identified an initial baseline group of apps by searching the Australian Google Play store for the Android platform (Google, Mountain View, CA, USA) and the Australian iTunes for the iOS platform (Apple Inc, Cupertino, CA, USA) using the keyword “depression.” Results were limited by the search engines to a maximum of 190 (Android) or 200 (iOS) apps. We also performed secondary searches for “bipolar disorder” and “suicide” to allow a comparison of the search result and app half-lives across other mental health domains as part of the primary aim; however, we did not consider them for clinical relevance in the secondary aim or for substantiation of effectiveness claims in the tertiary aim.

We created a custom script to automatically repeat the app store searches every day over a 9-month period. If an app identified on a previous day no longer appeared in the keyword search results, we performed an additional search for that specific app. This allowed a differentiation between an app that no longer appeared in the search results (eg, due to decreasing popularity) and an app that was no longer available (eg, having being withdrawn by the developer).

We linked longitudinal data for each app using its unique package or bundle identifier within the app store. This unique identifier allows multiple apps with the same name to be differentiated, as well as tracking of a single app if it is rebranded with a new name. Textbox 1 summarizes the data items recorded each day. We recorded user-rated quality through the average app store star rating, as well as the number of reviews that contributed to the average. For consistency across the Android and iOS app stores, we recorded the rating for the entire history of the app rather than the rating for just the current version, which was only available from the iOS store. The number of downloads was only available for Android apps and was reported as a broad category (<50, 50–100, 100–500, 500–1000, 1000–5000, 5000–10,000, 10,000–50,000, 50,000–250,000, and >250,000).

**Mental health app characteristic data collected each day from the Android and iOS app stores.**

•App available to download from app store (yes/no)

•App appears in keyword search results (yes/no)

•Search result ranking

•Version

•User rating (star rating)

•Number of user reviews

•Number of downloads (Android only)

**Figure 1:**
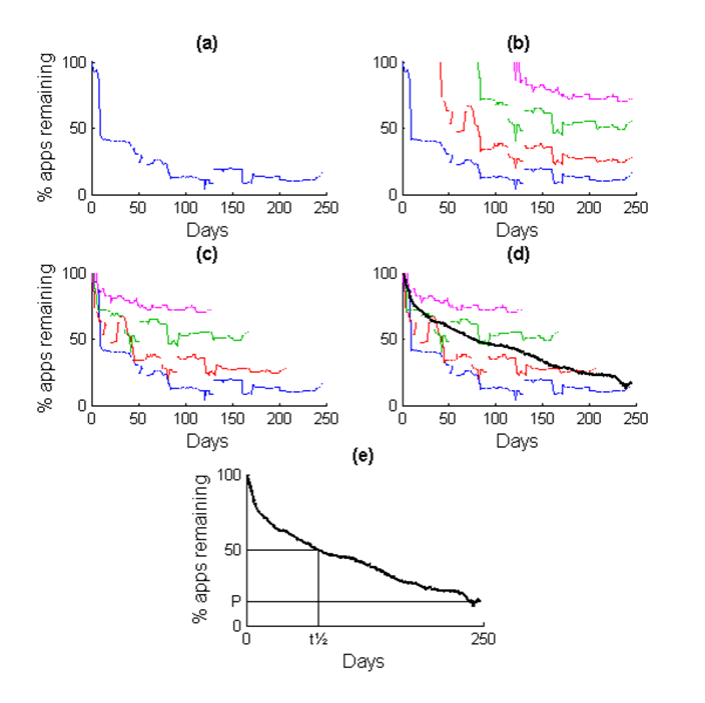
The process to calculate the search result half-life (t_½_). (a) A time series showing the proportion of apps that still appear in the search results each day, following the initial search on day 1. (b) This process is repeated for each day of the study, shown as different-colored time series (only 4 used to illustrate). (c) The time series shifted to begin at a common time point, such that the day of the search equals day 1. (d) The average of the time series was calculated (black line). (e) The t_½_, where the average time series crosses the 50% threshold, and the proportion of apps remaining on the final day of data collection were then calculated. The same process is used to calculate the app t_½_, where the y-axes represent the proportion of apps still available to download.

### Half-Life Calculation

Figure 1 illustrates the process for calculating the search result half-life. On day 1, we performed a keyword search of the app store and recorded the results. We calculated the proportion of these apps that also appeared in the search results for each subsequent day (Figure 1, part a). We then repeated this for each new search on each day of the study period (Figure 1, part b). Then we shifted each of these time series to a common reference point, such that the day the search was performed was denoted as day 1 (Figure 1, part c), and calculated the average of these time series (Figure 1, part d). We calculated the average values only if data from at least 20 days were available. Calculating the average in this way reduced the sensitivity to changes in the half-life period based on differences in the start of the data collection period, thereby providing a more generalized measure of the dynamics in the app stores. We calculated the search result half-life (t_½_) as the number of days after which the average number of apps still appearing in the search results dropped to 50% (Figure 1, part e). We also calculated the percentage of apps remaining on the last day of data collection. We applied a similar process to calculate the app half-life. In this case, the individual time series represented the proportion of apps that were still available to download—that is, if the app either appeared in the keyword search results, or was found by searching for its unique identifier. This contrasts with the search result half-life, which only included apps that appeared in the keyword search results.

### Screening for Clinical Relevance

We screened all of the apps identified during the data collection period for clinical relevance. The title and description of each app were independently assessed by 2 reviewers to identify apps related to depression or depressive symptomology, although they did not assess clinical quality or suitability for clinical recommendation. Results of the screening were compared, and discrepancies were resolved by discussion until consensus was achieved. The proportion of clinically relevant apps was calculated. Search result and app half-lives were also calculated separately for the subgroups of apps that were identified as clinically relevant or not clinically relevant.

### Identification of Effectiveness Claims

Apps that were identified as being clinically relevant for depression were assessed for claims of evidence. The apps were initially filtered by searching the app store descriptions for any of the following keywords: effective*, clinical, study, studies, proven, proof, evaluate*, tested, guaranteed, evidence, RCT [randomized controlled trial], or trial. Apps that matched at least one of these keywords were independently screened by 2 reviewers to identify whether the app store description provided evidence of any claims of effectiveness. Results of this screening were compared, and discrepancies were resolved by discussion until consensus was achieved. The proportion of apps providing evidence was then calculated.

### Supplementary Subgroup Analyses

We defined subgroups of apps based on publicly visible app store factors that may affect the search result and app half-lives. The first subgroups were defined as the top and bottom 25 apps in the search result rankings. Similar subgroups were defined based on the top and bottom apps in terms of number of reviews, star ratings, and number of downloads (Android only). The search result and app half-life measures for these subgroups were calculated and are reported in Multimedia Appendix 1.

## Results

We performed the initial search of the app stores on January 19, 2015 and repeated the search each day until September 21, 2015 (246 days). Data were not available for 35 days due to, for example, problems with network connectivity, resulting in a complete dataset for 211 days during this period. The maximum number of apps was returned for the “depression” keyword search on the first day, specifically 190 Android apps and 200 iOS apps (Table 1). Over the course of the study, 623 unique Android apps and 359 unique iOS apps appeared in the search results for the “depression” keyword. Table 1 also shows the results for the “bipolar disorder” and “suicide” searches.

**Table 1:** Number of unique apps identified on the first search day and through the data collection period.

Search term	Platform	Apps identified on first day (n)	Total apps (n)
Depression	Android	190	623
	iOS	200	359
Bipolar disorder	Android	159	535
	iOS	40	47
Suicide	Android	190	694
	iOS	144	206

### Search Result Half-Life

**Figure 2:**
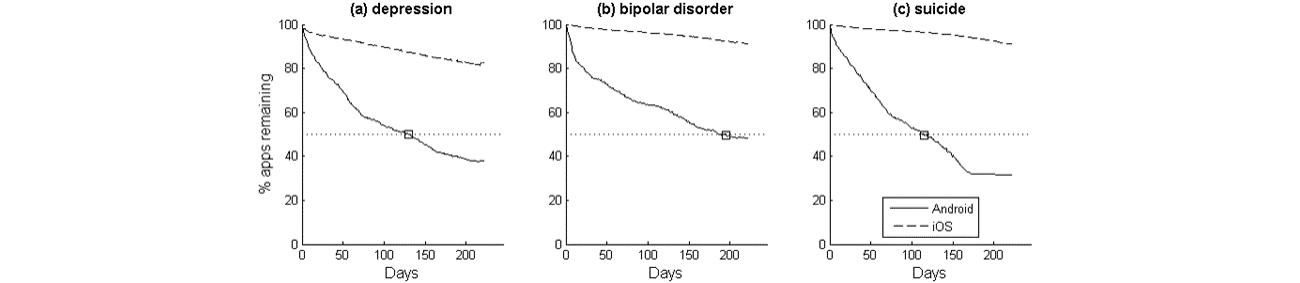
Average time series trends of apps remaining in the search results for (a) depression, (b) bipolar disorder, and (c) suicide. Crossing points with the 50% threshold are highlighted to indicate the search result half-life (t_½_).

Figure 2 shows the average time series reflecting the proportion of apps that remained in the search results in subsequent days for the 3 search terms. The trends showed general decreases in the apps that remained in the search results. On Android, the search result half-life for depression apps was t_½_=130 days, indicating that, on average, 50% of search results changed after 130 days. The search result half-lives for the Android bipolar disorder and suicide searches were 195 and 115 days, respectively. The proportion of search results remaining did not drop below 50% for any of the 3 search terms on iOS, indicating that the search result half-lives exceeded 9 months. Table 2 shows the full results.

**Table 2:** Search result half-life (t_½_) and the proportion of apps remaining in the search results at the end of the study.

Search term	Platform	Search result t_½_	Apps remaining at end of study
Depression	Android	130 days	37.8%
	iOS	>9 months	82.7%
Bipolar disorder	Android	195 days	48.4%
	iOS	>9 months	91.1%
Suicide	Android	115 days	31.6%
	iOS	>9 months	91.2%

### App Half-Life

**Figure 3:**
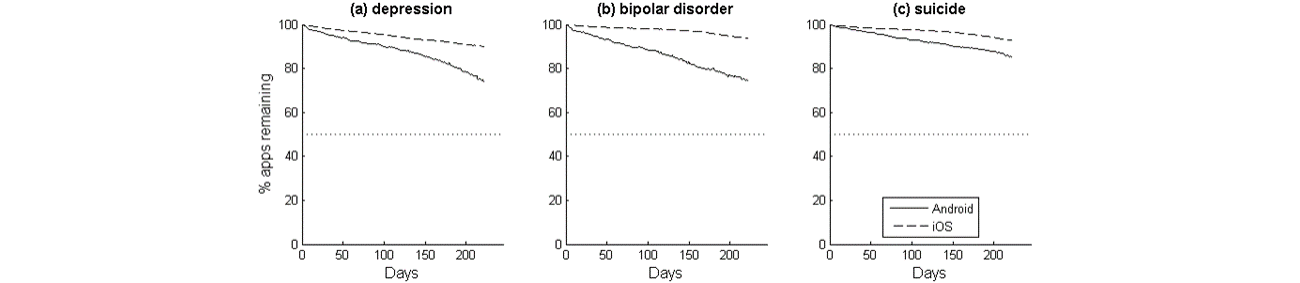
Average time series trends of apps still available to download from the app store, for (a) depression, (b) bipolar disorder, and (c) suicide.

Figure 3 shows the average time series of the proportion of apps still remaining in the app stores. None of the search terms on either platform crossed the 50% threshold, indicating that the app half-lives exceeded 9 months for each of the search terms, on both platforms. This indicates that, although apps may have disappeared from the search results, many continued to be available for download. Table 3 shows the full results.

**Table 3:** App half-life (t_½_) and the proportion of apps still available to download from the app stores at the end of the study.

Search term	Platform	App t_½_	Apps remaining at end of study
Depression	Android	>9 months	74.2%
	iOS	>9 months	90.0%
Bipolar disorder	Android	>9 months	74.4%
	iOS	>9 months	93.6%
Suicide	Android	>9 months	85.2%
	iOS	>9 months	92.9%

### Clinical Relevance

**Figure 4:**
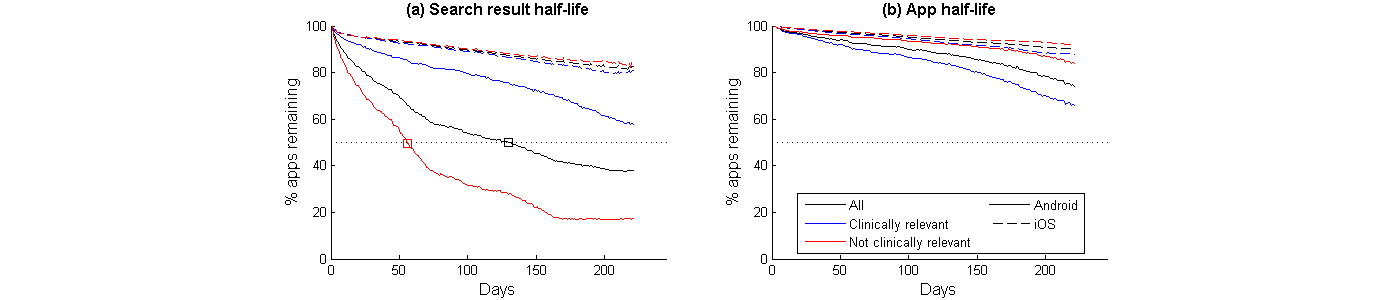
Average time series trends of (a) apps remaining in the search results, and (b) apps still available to download. Plots are shown for all apps on each platform and grouped by whether they were clinically relevant to depression. Crossing points with the 50% threshold are highlighted to indicate the search result half-life (t_½_).

Of the 623 Android apps identified in the depression searches, we screened 197 (31.6%) as being clinically relevant to the condition. On the iOS platform, we identified 150 of the 359 apps (41.8%) as relevant. Figure 4 and Table 4 summarize the search result and app half-lives for the subgroups of relevant and nonrelevant apps.

**Table 4:** Search result half-life (t_½_), app half-life, and the proportion of apps still available to download from the app stores at the end of the study, grouped by whether they were clinically relevant to depression.

Platform	Clinical relevance subgroup	Search result		Available to download	
		t_½_	Apps remaining	t_½_	Apps remaining
Android	All	130 days	37.8%	>9 months	74.2%
	Relevant	>9 months	57.9%	>9 months	65.9%
	Not relevant	56 days	17.2%	>9 months	83.8%
iOS	All	>9 months	82.7%	>9 months	90.0%
	Relevant	>9 months	80.7%	>9 months	87.8%
	Not relevant	>9 months	84.4%	>9 months	91.9%

There was very little difference between the iOS apps identified as being clinically relevant and those that were not clinically relevant. A similar proportion remained in the search results after 9 months (80.7% vs 84.4%) and were still available to download (87.8% vs 91.9%). The difference was greater in the Android apps, where more clinical apps remained in the search results (57.9% vs 17.2%), although fewer remained available for download (65.9% vs 83.8%).

### Identification of Effectiveness Claims

Across both the Android and iOS platforms, we identified 347 apps as being clinically relevant for depression. We manually screened the 131 of these apps that matched at least one of the keywords related to claims of effectiveness. Of the 347 clinically relevant apps, 9 (2.6%) provided some degree of evidence to support their claims of effectiveness: only 3 apps included a full citation to a study; 3 apps included a partial citation (eg, author and year only); 2 mentioned an unspecified and unreferenced study; and 1 included a link to a website purportedly containing evidence, which we were not able to access at the time of the review.

## Discussion

To our knowledge, this is the first examination of the dynamics of app marketplaces, and particularly with a focus on mental health apps. The results indicate that the mental health app environment changes daily. Volatility exists in both the visibility of apps, with half the search results changing within approximately 4 months, and in the continued availability to download apps. The number of clinically relevant apps that were no longer available to download at the end of the study period was equivalent to a depression app disappearing every 3.7 days on Android, every 13.7 days on iOS, or every 2.9 days across both platforms.

Interestingly, both the appearance in search results and continued download availability were more stable in the iOS app store than in the Android store. This is possibly partly due to a greater number of apps in the Android store; therefore, the search engine can display only a subset of the apps, increasing the likelihood of apps rising into, and falling out of, the search results. However, this does not entirely explain our observation, as the results for bipolar disorder were not truncated, but Android apps still had a shorter half-life than their iOS counterparts. The formal app review process undertaken by Apple before making an app available in their store may contribute to this finding.

Over the study period, fewer than half the apps identified using the “depression” keyword were clinically relevant to the disorder. This presents a challenge for mHealth utility and confirms the problematic indexing and searching of mental health apps highlighted previously [11,12]. Shen et al [12] reported that one-fifth of the results of a search for “depression” in the Google, Apple, Windows, Nokia, and BlackBerry app stores did not mention depression in the title or app description, and 75% of the results were not relevant to consumers with the condition. This may be partly due to ambiguity in indexing app descriptions and to developers seeking to increase the visibility of their apps [13].

This volatility presents a challenge for consumers or clinicians seeking apps for mental health conditions, as rapidly changing search results and app availability, along with irrelevant search results, may affect confidence in this technology to facilitate and extend mental health treatment and support. With approximately one-third of clinically relevant depression Android apps no longer being available after 9 months, clinicians are in danger of recommending apps that are no longer available, and consumers may be left with defunct apps, no longer supported by the developer and without critical updates needed for continued use. Across both the Android and iOS platforms, a quarter of clinically relevant apps were no longer available after the 9-month study period. This rate of turnover is faster than that reported by Huckvale et al [9] in their updated review of asthma apps, which found that a similar proportion were no longer available after a period of 2 years.

The combination of changing search results and uncertain app availability adds to the challenge faced by consumers in identifying relevant mental health apps. To increase the relevance of the search results, searches could be performed within specific categories of apps. Developers assign their apps to predetermined app store categories, but there is no clear way for consumers to search for a keyword within these categories. Such a capability would assist in the disambiguation of terms, for example, clinical depression (a medical app) versus the great depression (a financial app).

The challenge of finding a relevant mental health app is further confounded by the absence of information about app effectiveness and a lack of substantiation of any such claim. Only 2.6% of depression apps identified in this review attempted to substantiate claims of effectiveness. This is lower than the 15% of apps that Leigh and Flatt previously identified as providing evidence for effectiveness [6]; however, this is not surprising considering the apps in their review had been endorsed for inclusion in the NHS Health Apps Library. One possible method of encouraging evaluation and reporting of clinical effectiveness would be for the app stores to allow the inclusion of a PubMed article identifier, allowing users to click through to published articles related to the app. The limited information on clinical effectiveness in app descriptions results in consumers and clinicians basing app choice on incomplete or potentially incorrect information.

There have therefore been calls for systems to assist consumers with app selection, which have primarily focused on the development of app quality indicators. However, this has proven to be difficult. Issues have plagued numerous attempts at app accreditation portals. Privacy and security flaws were uncovered in apps approved by both the Happtique and NHS Health Apps Library accreditation sites, both now offline [9,13]. A recent paper has also demonstrated obstacles with user-based app rating tools: Powell et al [14] demonstrated poor interrater reliability among practitioners rating mental health apps. Wicks and Chiauzzi [15] suggested another solution that places quality assurance at the consumer point-of-contact—specifically, the app stores. However, due to the extensive resources required to assess the health-related content of each submitted app, it is unlikely that app store operators will be able to provide this service. Nevertheless, changes in indexing, store descriptions, and search algorithms would improve search result stability and consumer experience, making relevant apps easier to find and select.

### Implications for Systematic Reviews of App Content

This study also highlights a challenge for researchers evaluating and reviewing apps available for psychiatric disorders, as such volatility affects the reproducibility and the medium- to long-term validity of findings. Such app reviews provide a snapshot of the marketplace, but also can be a resource for clinicians to find information about the quality of available apps.

The range of mental health apps available in the iOS app store was relatively stable over a period of 9 months. Researchers reviewing apps can be confident that the body of apps reviewed will still be representative after 9 months, and likely over a year.

The Android app store is more dynamic: one-third of depression-related apps could not be downloaded after 9 months, which poses additional challenges. Researchers conducting reviews of apps must therefore be mindful that the results will become at least partially outdated during the duration of the review process. The rapid rate of change indicates that a rereview would be required two to three times a year to remain current. Given the impracticability of this schedule, we suggest that reviewers (1) clearly indicate the date on which searches are performed, (2) perform an updated search prior to final submission of a review manuscript, and (3) indicate in the final manuscript or supplementary material which apps are still available.

### Limitations

A possible limitation of this study was the narrow scope of search terms included. The 3 terms selected (depression, bipolar disorder, and suicide) cannot capture the full spectrum of mental health apps available, nor provide a comprehensive set of apps for specific mental health conditions. The wider applicability of the results for physical health apps is also uncertain. We therefore recommend using a wider set of keywords for searches in future studies. The searches were also limited to the two most popular app stores, for Android and iOS platforms. These results may therefore not be representative of apps for Windows or BlackBerry devices, or apps downloaded through unofficial stores.

A second possible limitation is that the criteria used to screen apps were relatively simplistic, focusing on relevance for one specific condition. We did not assess app quality or the evidence quality of any claims of effectiveness; therefore, the subgroup of clinically relevant apps may not exactly reflect the range of apps that would be recommended in routine practice.

In this study, we tracked apps longitudinally using their unique package or bundle identifier. While this allows tracking of multiple apps with the same name, as well as of individual apps that change names, it does not allow identification of apps that are rereleased with a new identifier. These would appear as distinct apps within the app store and a consumer’s handset. Additional in-app content analysis may allow identification of whether an app is relaunched in this manner.

The results of the subgroup analyses are provided in Multimedia Appendix 1; however, these results should be interpreted with caution. This is especially true considering the small sample sizes in the groups (25 in the top vs bottom comparisons), as spurious results from such analyses are possible [16].

### Conclusions

Our study highlights challenges with the app store environments in which mobile apps operate that need to be addressed. The changing nature of the app marketplace, combined with the predominance of nonrelevant search results and lack of effectiveness indicators, may affect the ability of mHealth to fulfill expectations. Future studies may seek to further understand the reasons for app turnover, considering the factors publicly available from app store search results and further content analysis of in-app features. With 50% of search results changing within 4 months and an app being removed every 2.9 days, the mHealth space presents a challenge for consumers, particularly in the absence of evaluative resources. As such, consumers looking to use apps for mental health should consider the following during app selection: in the absence of app effectiveness information, determine the effectiveness of the app’s approach, rather than of the app itself; note the developer of the app, in particular their expertise and reputation in mental health; and examine the update history of the app to gauge whether the developer is still engaged. Ultimately, this form of consumer education about the app store and app use is needed to increase app literacy and reduce the impact of the app store environment until it is addressed.

## Appendix

Multimedia Appendix 1 Subgroup analyses.

## Abbreviations

NHS: National Health Service

## References

[1] IDC (2013). Always Connected: How Smartphones and Social Keep Us Engaged.

[2] Comstock J (2014). Survey: 32 Percent of Mobile Device Owners Use Fitness Apps.

[3] Torous J, Chan SR, Yee-Marie TS, Behrens J, Mathew I, Conrad EJ, Hinton L, Yellowlees P, Keshavan M (2014). Patient smartphone ownership and interest in mobile apps to monitor symptoms of mental health conditions: a survey in four geographically distinct psychiatric clinics. JMIR Ment Health.

[4] Ericsson Mobility Report (2015). On the Pulse of the Networked Society.

[5] Phillips S (2015). App Stores Will be the Number One Distribution Channel for mHealth Apps Until at Least 2020.

[6] Leigh S, Flatt S (2015). App-based psychological interventions: friend or foe?. Evid Based Ment Health.

[7] Donker T, Petrie K, Proudfoot J, Clarke J, Birch M, Christensen H (2013). Smartphones for smarter delivery of mental health programs: a systematic review. J Med Internet Res.

[8] Torous J, Powell AC (2015). Current research and trends in the use of smartphone applications for mood disorders. Internet Intervent.

[9] Huckvale K, Morrison C, Ouyang J, Ghaghda A, Car J (2015). The evolution of mobile apps for asthma: an updated systematic assessment of content and tools. BMC Med.

[10] Larsen ME, Nicholas J, Christensen H (2016). A systematic assessment of smartphone tools for suicide prevention. PLoS One.

[11] Nicholas J, Larsen ME, Proudfoot J, Christensen H (2015). Mobile apps for bipolar disorder: a systematic review of features and content quality. J Med Internet Res.

[12] Shen N, Levitan M, Johnson A, Bender JL, Hamilton-Page M, Jadad AA, Wiljer D (2015). Finding a depression app: a review and content analysis of the depression app marketplace. JMIR Mhealth Uhealth.

[13] Boudreaux ED, Waring ME, Hayes RB, Sadasivam RS, Mullen S, Pagoto S (2014). Evaluating and selecting mobile health apps: strategies for healthcare providers and healthcare organizations. Transl Behav Med.

[14] Powell AC, Torous J, Chan S, Raynor GS, Shwarts E, Shanahan M, Landman AB (2016). Interrater reliability of mHealth app rating measures: analysis of top depression and smoking cessation apps. JMIR Mhealth Uhealth.

[15] Wicks P, Chiauzzi E (2015). 'Trust but verify': five approaches to ensure safe medical apps. BMC Med.

[16] Sleight P (2000). Debate: Subgroup analyses in clinical trials: fun to look at - but don't believe them!. Curr Control Trials Cardiovasc Med.

