# Supplementary material for: Quantifying App Store Dynamics: Longitudinal Tracking of Mental Health Apps
Source: JMIR Mhealth Uhealth. 2016 Aug 9;4(3):e96. doi: 10.2196/mhealth.6020 (PMC4995352; doi:10.2196/mhealth.6020)
Supplement: Multimedia Appendix 1 [file mhealth_v4i3e96_app1.pdf]

Multimedia Appendix 1: Sub-group analyses

Four sub-group analyses were defined to allow a comparison between: (a) the top and bottom 25 apps in the search result rankings, (b) the top and bottom 25 apps in terms of number of reviews, (c) the top and bottom 25 apps in terms of star-ratings, and (d) the top and bottom 25 apps in terms of the number of downloads (Android only).

User reviews were available for 533 of the Android depression apps (85.6%), with between 1 and 5.64 million reviews (median: 379). The median score of these reviews was 4.1 stars. On iOS, reviews were available for 78 depression apps (21.7%), with between 5 and 806 reviews per app (median: 16). The median app rating was 4.0 stars. Eighty-nine Android apps for depression (14.3%) had been downloaded fewer than 50 times, and 185 (29.7%) had been downloaded over 250,000 times. The median number of downloads lay in the range 10,000-50,000.

The results of the sub-group analyses of the depression apps are shown in Figure 5 (search result half-life) and Figure 6 (app half-life). These results are also summarised in Table 5.

Search result ranking

Comparing apps which appeared in the top 25 and bottom 25 search results, there was relatively little difference in the trajectory of app availability to download on either platform (82.6% vs 80.8% on Android, and 96.0% vs 92.6% on iOS). Search result position did, however, have a large impact on retention in the search results. On Android 82.4% of the top 25 apps remained in the search results after nine months, compared to just 7.48% of the bottom 25 apps. A similar, but smaller, effect was observed for iOS apps, with 95.8% and 58.8% of the top and bottom 25 apps, respectively, remaining in the search results.

Number of reviews and star ratings

No clear pattern was observed in the sub-group analysis based on the number of reviews. Apps with fewer reviews tended to become unavailable to download on the Android app store (88.1% compared to 49.5%), although the opposite effect was observed in the iOS store (88.0% compared to 97.8%). On both platforms, apps with fewer reviews remained in the search results longer than those with more reviews. There was a tendency for apps with higher star-ratings both to remain available to download, and to remain visible in the search results for a longer period. However, the opposite was true for most of the study period on

Android, where apps with lower star ratings were more likely to remain in the search results than apps with higher star ratings - as shown in Figure 5(c).

Number of downloads

The number of downloads per app was not available on iOS, however there is no clear pattern in the sub-group analysis for the Android platform. Although apps which had been downloaded more often tended to remain on the app store longer than those with fewer downloads, those with more downloads remained in the search results for a shorter period.

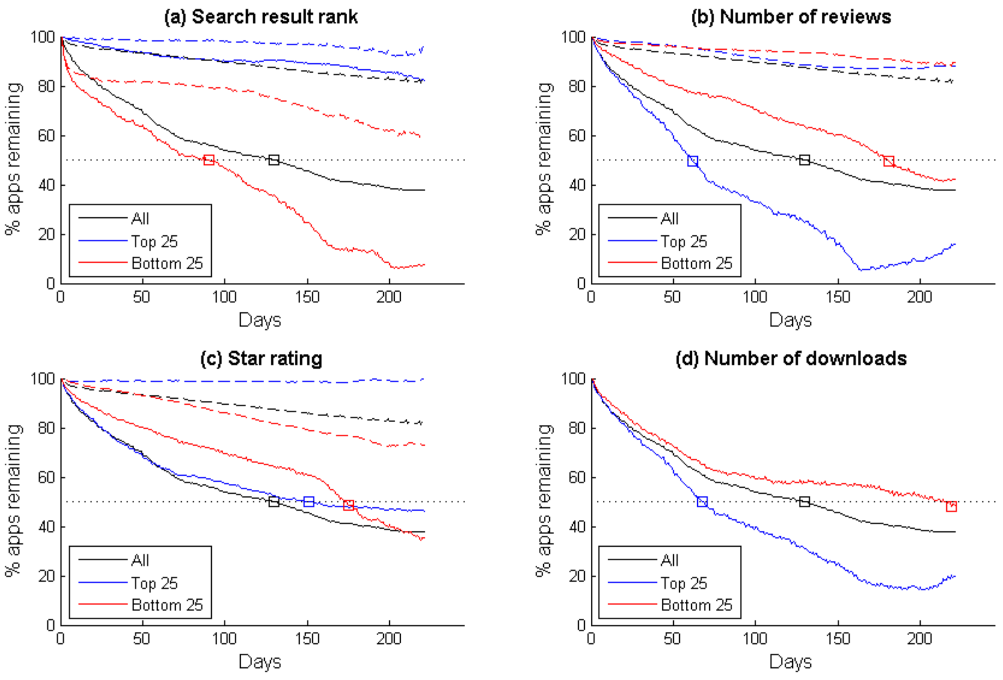

Figure 5 – Average time series trends of app sub-groups remaining in the search results. Subgroups are based on (a) ranking within search results; (b) number of reviews for each app; (c)average rating for each app; and (d) number of downloads (Android only). Solid lines represent the Android platform, and dashed lines represent iOS. Crossing points with the 50% threshold are highlighted to indicate the search result half-life ($t_{½}$).

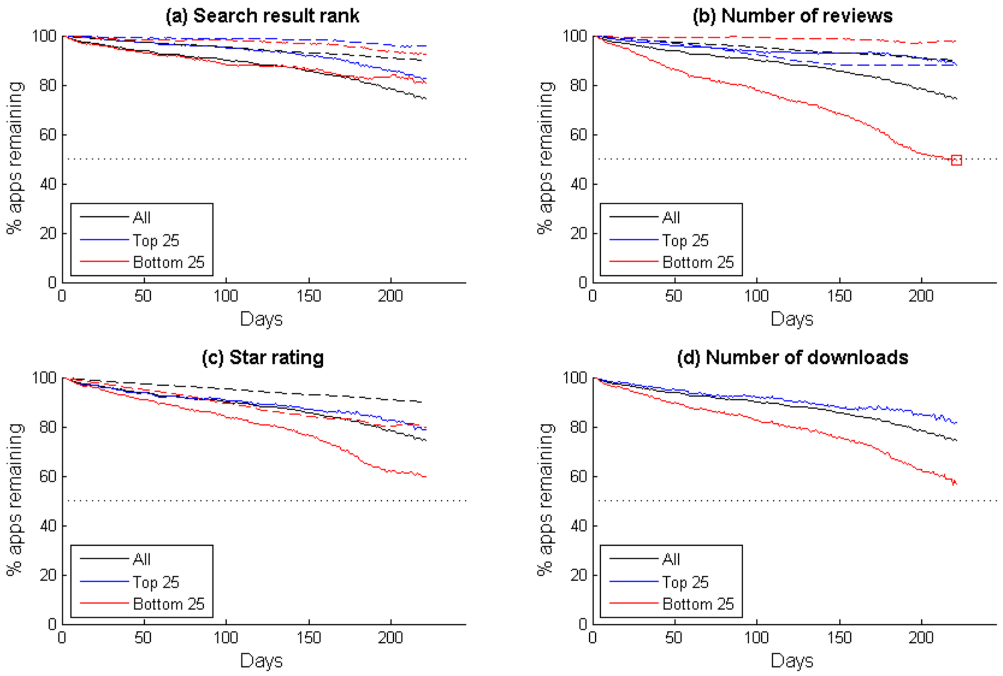

Figure 6 – Average time series trends of app sub-groups remaining available for download. Subgroups are based on (a) ranking within search results; (b) number of reviews for each app; (c)average rating for each app; and (d) number of downloads (Android only). Solid lines represent the Android platform, and dashed lines represent iOS. Crossing points with the 50% threshold are highlighted to indicate the app half-life ($t_{½}$).

Table 5 – Sub-group analyses of search result half-life, app half-life, and the proportions of apps remaining in the search results at the end of the study. Sub-groups are based on human screening for clinical relevance, search result ranking, number of reviews, star-rating in reviews, and number of downloads.

Platform	Category	Sub-group	Search Result		Available to download	
			$t_{½}$	Apps remaining	$t_{½}$	Apps remaining
Android	All	All	130 days	37.8%	>9 months	74.2%
	Clinical relevance	Relevant	>9 months	57.9%	>9 months	65.9%
		Not relevant	56 days	17.2%	>9 months	83.8%
	Search result rank	Top 25	>9 months	82.4%	>9 months	82.6%
		Bottom 25	90 days	7.48%	>9 months	80.8%
	Number of reviews	Top 25	62 days	16.0%	>9 months	88.1%
		Bottom 25	181 days	42.1%	221 days	49.5%
	Star rating	Top 25	151 days	46.0%	>9 months	78.6%
		Bottom 25	175 days	35.5%	>9 months	59.7%
	Number of downloads	Top 25	68 days	19.8%	>9 months	81.6%
		Bottom 25	219 days	48.6%	>9 months	56.8%
iOS	All	All	>9 months	82.7%	>9 months	90.0%
	Clinical relevance	Relevant	>9 months	80.7%	>9 months	87.8%
		Not relevant	>9 months	84.4%	>9 months	91.9%
	Search result rank	Top 25	>9 months	95.8%	>9 months	96.0%
		Bottom 25	>9 months	58.8%	>9 months	92.6%
	Number of reviews	Top 25	>9 months	88.0%	>9 months	88.0%
		Bottom 25	>9 months	89.4%	>9 months	97.8%
	Star rating	Top 25	>9 months	99.8%	>9 months	100.0%
		Bottom 25	>9 months	72.8%	>9 months	79.6%
